# The role of sex differences in depression in pathologically defined Alzheimer’s disease

**DOI:** 10.3389/fnagi.2023.1156764

**Published:** 2023-05-10

**Authors:** Cécilia Tremblay, Parichita Choudhury, Christine M. Belden, Danielle Goldfarb, Ileana Lorenzini, Thomas G. Beach, Geidy E. Serrano

**Affiliations:** Department of Neuropathology, Banner Sun Health Research Institute, Sun City, AZ, United States

**Keywords:** gender, neuropsychaitric symptoms, behavioral and psychiatric symptoms of dementia, neuropathology, postmortem, sex differences, depression, Hamilton rating scale for depression

## Abstract

**Introduction:**

Sex differences in Alzheimer’s disease (AD) may contribute to disease heterogeneity and affect prevalence, risk factors, disease trajectories and outcomes. Depression impacts a large number of patients with AD and has been reported to be more prevalent in women. We aimed to better understand the interaction between sex, depression and AD neuropathology, which could have implications for detection of symptoms, earlier diagnosis, therapeutic management, and enhanced quality of life.

**Methods:**

We compared 338 cases with clinicopathologically confirmed AD (46% women) to 258 control cases (50% women), without dementia, parkinsonism or a significant pathological diagnosis. Depression was assessed both, using the Hamilton Depression Scale (HAM-D), and as being reported in their medical history combined with treatment with antidepressant medication.

**Results:**

In the control group, women showed a higher depression severity, and a higher proportion of women were found to meet the cut-off score for depression on the HAM-D (32 vs. 16%) and having an history of depression (33 vs. 21%), while these sex differences were not observed in AD. Further, in both groups, female sex independently predicted the presence of depression, with covariates for age and cognitive status. AD subjects had higher mean HAM-D scores, were more likely to meet cutoff scores for depression (41 vs. 24%) and have a history of depression than controls (47 vs. 27%). When comparing the increase in frequency of depression in controls versus AD, the difference was significantly greater in men (AD men - control men: 24%) than in women (AD women - control women: 9%). Although subjects with depression were more likely to have higher levels of AD neuropathology, these differences were not observed when investigating the control or AD group separately.

**Discussion:**

Control women had a higher likelihood and severity of depression than control men, but this sex difference was not noted when considering only those with pathologically defined AD, emphasizing the importance of considering sex in aging studies. AD was associated with higher rates of depression and men may be more likely to report or be diagnosed with depression once they develop AD indicating the importance of more frequent depression screenings in men.

## 1. Introduction

Alzheimer’s disease (AD) is the most common neurodegenerative disease and cause of dementia. AD is associated with progressive decline in memory, executive, and other cognitive functions leading to functional decline and can manifest with diverse clinical presentations and heterogeneity (Murray et al., 2011; Atri, 2019; Knopman et al., 2021). Sex differences in AD have been shown to contribute to this heterogeneity and affect prevalence, risks factors, disease trajectories, and outcomes (Nebel et al., 2018; Katabathula et al., 2022). Depression is a frequent neuropsychiatric symptom that affects many patients with AD and has been shown to be more prevalent in women in the general population as well as in dementia (Lovheim et al., 2009; Chi et al., 2015). Therefore, a better understanding of the neuropathology of sex differences in depression among patients with AD may offer insights into strategies for prevention, diagnosis, therapy, and quality of life (Ferretti et al., 2018).

Sex differences in AD have been previously reported in terms of epidemiology, symptomatology, progression, risk factors, and biomarkers of AD (Zhu et al., 2021). The proportion of women with clinical AD is substantially higher than for men, and although sex differences in the risk of developing AD have yielded mixed findings, AD was found to be more prevalent in women at older ages (Mielke et al., 2014; Rocca, 2017; Ferretti et al., 2018; Zhu et al., 2021). Women were also shown to demonstrate a faster cognitive decline and greater clinical and pathological severity (Filon et al., 2016; Koran et al., 2017; Barnes et al., 2019; Zhu et al., 2021). Therefore, more studies are needed to better understand the effect of sex on disease heterogeneity and how they can be used in profiling disease phenotypes (Cahill, 2006; Filon et al., 2016; Liesinger et al., 2018; Nebel et al., 2018; Buckley et al., 2019).

Depression is one of the most common psychiatric disorders and impacts a large number of patients with AD, with studies reporting between 20 to 60% of patients affected with depression (Lyketsos and Olin, 2002; Chi et al., 2015; Moustafa et al., 2022). Depression considerably undermines the quality of life in patients and their caregivers, increases caregiver burden and aggravates psychological pain. In addition, depression itself has been suggested to be a risk factor of AD as well as a predictor of cognitive decline (Tsuno and Homma, 2009; Panza et al., 2010; Barnes et al., 2012; Diniz et al., 2013; Hudon et al., 2020; Anstey et al., 2021; Saiz-Vazquez et al., 2021). Sex differences have been identified in depression, with a higher prevalence and a greater illness severity observed in women in the general population (Parker and Brotchie, 2010; Salk et al., 2017; Labaka et al., 2018; Eid et al., 2019; Bangasser and Cuarenta, 2021). In AD dementia, women were also more likely to have depressive symptoms, however, studies have yielded inconsistent results (Lovheim et al., 2009; Lee et al., 2017; Tao et al., 2018; Eikelboom et al., 2022). Moreover, inconsistent findings have been reported regarding the influence of sex in the association of depression as a risk factor for AD (Underwood et al., 2019; Zhu et al., 2021). These discrepancies may be related to the use of different tests to diagnose depression and difficulties in diagnosing depression in AD due to the presence of several neuropsychiatric symptoms, overlapping symptoms between depression and dementia, the lack of consensus criteria to diagnose depression in AD and relying on the therapeutic metaphor with discrete psychiatric disorders (Tariot, 1999; Lyketsos et al., 2011; Novais and Starkstein, 2015; Burke et al., 2019; Moustafa et al., 2022). Further, all these studies lack pathological confirmation of an AD diagnosis which creates additional uncertainties as dementia in older individuals may be related to non-AD or mixed pathologies in the brain (Beach and Malek-Ahmadi, 2021).

Hence, this study aimed to investigate sex differences in depression, comparing both measures of depression from a validated scale scored test as well as depression reported in a subject’s medical history combined with treatment with anti-depressant medication, and its link to neuropathology of AD in a well-characterized group of subjects with cognition ranging from unimpaired to dementia, derived from a longitudinal clinicopathological study.

## 2. Methods

### 2.1. Subjects

Subjects included in this study were volunteers enrolled in the Arizona Study of Aging and Neurodegenerative Disorders (AZSAND) and Brain and Body Donation Program (BBDP; www.brainandbodydonationprogram.org), a longitudinal clinicopathological study at Banner Sun Health Research Institute (BISHRI) in metropolitan Phoenix, Arizona (Beach et al., 2015). All subjects signed informed consents, approved by BSHRI Institutional Review Boards, for both clinical assessment and brain donation for research purposes. Subjects are clinically characterized with annual standardized test batteries, consisting of general neurological, cognitive, and movement disorders components that are assessed by cognitive/behavioral neurologists, movement disorders neurologists and neuropsychologists (Beach et al., 2008, 2015).

Subjects of the current study were chosen by searching the BBDP database for cases that were assigned a final clinicopathological consensus diagnosis of AD dementia or control (defined as clinically lacking dementia or parkinsonism); these subjects could have mild cognitive impairment or incidental pathology but did not meet criteria for clinical or pathological diagnosis of a neurodegenerative disease (Beach et al., 2015). All subjects completed at least one clinical assessment of symptoms of depression using a scaled validated tool, before death. Data available included global cognition assessment using the Mini-Mental State Examination (MMSE) as well as the age of onset of dementia symptoms, which was used to calculate the duration of dementia. A total of 596 cases, between 2001 and 2021, including 338 (157 women/181 men) cases with AD and 258 (131 women/ 127 men) control cases were included in this study. The age at death of subjects ranged from 56 to 104 years with a mean age of death of 87 years (report to Table 1).

**Table 1:** General, cognitive, and depression-related characteristics of all study subjects divided by group and sex.

	Controls (258)		AD (338)	
	Women (131)	Men (127)	Women (157)	Men (181)
Age at death	88.8 (6.9)	87.6 (6.4)	87.7 (7.7)	85.4 (7.2)* ^#^
Dementia, age onset			81.8 (8.4)	79.2 (8.3)*
Dementia, duration			6.2 (4.1)	6.0 (4.3)
MMSE	27.7 (2.2)	27.4 (2.2)	16.2 (8.8)	15.8 (8.2)^#^
Education	14.8 (2.5)	15.1 (2.9)	14.0 (2.5)	15.3 (2.8)* ^#^
HAM-D	6.2 (4.7)	4.7 (3.3)*	7.1 (4.7)	7.0 (4.4)^#^
GDS	4.5 (3.7)	3.9 (3.2)	4.2 (3.3)	3.9 (3.2)
NPI-Q	3.1 (3.6)	2.7 (3.8)	7.2 (5.1)	8.2 (5.6)^#^

Data are presented as means (SD). MMSE = last Mini Mental State Examination score; HAM-D = Hamilton Rating Scale for Depression; GDS = Geriatric Depression Scale (missing data for 203 subjects); NPI-Q = Neuropsychiatric Inventory Questionnaire (missing data for 152 subjects).

* *p* < 0.05 for sex comparisons within AD subjects or controls subjects.

# *p* < 0.05 for comparison of all 4 groups.

### 2.2. Depression assessments:

Different assessment scales for depression have been used over the years in BBDP including the clinician-administered Hamilton Rating Scale for Depression (HAM-D; Hamilton, 1960), the Geriatric Depression Scale (GDS; Yesavage et al., 1982) and depressive symptom assessment as part of the Neuropsychiatric Inventory Questionnaire (NPI-Q; Cummings et al., 1994). Depression tests were administered by neuropsychologists and trained psychometrists. Of the 596 cases included, 590 (99%) had a least one assessment for the HAM-D and 409 (68.6%) had at least one assessment using the GDS while 444 (74.5%) subjects received at least one NPIQ assessment. As the HAM-D was the most commonly used scale for depression in our program, we used the results from HAM-D (Total possible score = 52) for statistical analysis, but also reported mean results from other depression scales. If more than one test was performed before death, the worst score was used to capture any symptoms of depression. To assess the presence of clinical depression, previously recommended cut-off criteria for HAM-D were used as follows; a score between 0 to 7 indicated no depression; 8 to 17 mild depression, 18 to 24 moderate depression, and a score over 24 indicated severe depression (Cusin et al., 2009; Romera et al., 2011; Zimmerman et al., 2013). Moreover, any previous diagnosis of depression and use of antidepressants are recorded as part of a subject’s medical history, using the medical history questionnaire performed at each BBDP visit or from the private medical records obtained. Therefore, in addition to scale scored depression, the combination of a medical history of depression and treatment with antidepressants was also used to define depression in a second set of analyses. Medication included selective serotonin reuptake inhibitors (SSRIs), Serotonin and norepinephrine reuptake inhibitors (SNRIs), nortriptyline, mirtazapine and trazodone.

### 2.3. Neuropathological evaluation

A complete neuropathological examination was performed after death, as previously described (Beach et al., 2015). Assignments for AD Braak neurofibrillary (NF) stages (Braak et al., 2006), CERAD neuritic plaque density score (Mirra et al., 1991), Thal amyloid phase for Aβ plaque brain distribution (Thal et al., 2002), and alpha-synuclein (aSyn) stage according to the Unified Staging System for Lewy Body Disorders (USSLBD; Beach et al., 2009). Data also included regional and summary cortical brain density measures for tau neurofibrillary (NF) tangle and plaque load (for both, there is a total possible score of 15 based on summary of 0–3 scores in each of 5 regions: frontal association cortex, parietal association cortex, temporal association cortex, hippocampus CA1, and entorhinal/transentorhinal areas). Neuropathological AD diagnoses were defined as having “intermediate” or “high” criteria according to the National Institute on Aging/Reagan Institute criteria combined with a history of clinical dementia (Nia and Group, R. I. W, 1997; Hyman et al., 2012; Montine et al., 2012). Some AD subjects (183 cases; 30.7% of which 55% were men and 45% women) had additional comorbid neuropathologically-diagnosed conditions, including Parkinson’s disease (PD; *n* = 41), dementia with Lewy bodies (DLB; *n* = 56), vascular dementia (VaD; *n* = 73), progressive supranuclear palsy (PSP; *n* = 31), frontotemporal lobar degeneration with TDP-43 proteinopathy (FTLD-TDP; *n* = 9), and corticobasal degeneration (CBD; *n* = 1). These subjects were grouped as a “multiple diagnoses” group and were excluded in a second set of analyses to account for the influence of comorbid (non-AD) brain disease.

### 2.4. Statistical analyses

Statistical analyses were performed using SPSS software (IBM SPSS Statistics 23.0). Non-parametric Mann–Whitney U-test, Kruskal Wallis ANOVA with Bonferroni *post hoc* comparisons, ANCOVA, as well as chi-square test or Fisher exact test were used, as appropriate, for group and sex comparisons. Non-parametric Spearman correlations were used to assess correlations between depression severity scores and clinical and neuropathologic characteristics. A series of logistic regressions, with depression as the dependent variable (binary: presence or not of depression), was then performed to assess if sex was a predictor of depression, with covariates for age and MMSE as well as neuropathologic characteristics.

## 3. Results

Refer to Table 1 for basic demographic and clinical characteristics and Table 2 for neuropathologic characteristics of the cases included for both women and men in each group. Of the included subjects, 48.3% were women and 51.7% were men while 54.5% of women and 58.8% of men had a final clinicopathological diagnosis of AD, these differences were not significant (NS). Age at death was significantly higher in control subjects than in AD subjects (88.2 ± 6.7 vs. 86.5 ± 7.5; U = 49.6; *p* = 0.004).

**Table 2:** Neuropathological characteristics of all study subjects divided by group and sex.

	Controls (258)		AD (338)	
	Women (131)	Men (127)	Women (157)	Men (181)
Brain weight	1112.7 (105.5)	1247.1 (114.4)*	1055.8 (109.6)	1188.7 (118.9)* ^#^
Braak stage	3.5 (0.8)	3.1 (0.9)	4.8 (0.9)	4.7 (0.9)^#^
Plaque density	1.7 (1.2)	1.4 (1.2)	2.9 (0.2)	2.9 (0.3)^#^
Total brain NF load	5.9 (2.3)	5.5 (2.7)	10.6 (3.8)	10.3 (3.6)^#^
Total brain plaque load	6.8 (5.6)	5.6 (5.7)	12.7 (2.8)	13.0 (2.3)^#^

Data are presented as means (SD).

* *p* < 0.05 for sex comparisons within AD or controls subjects.

# *p* < 0.05 for comparison of all 4 groups.

Age at death was significantly greater in women (88.2 ± 7.4 vs. 86.3 ± 6.3; U = 51.55; *p* < 0.001) and age at onset of dementia was also higher in women (81.8 vs. 79.2; U = 16.20; *p* = 0.002). Men had more education than women (U = 35.81; *p* < 0.001). No sex differences were found for duration of dementia, or last MMSE test score (U = 46.8 *p* = 0.08) prior to death. Apathy, as measured though the NPI-Q, was significantly more frequent and severe (U = 11808.0; *p* = 0.001) in AD than in controls (69% in AD vs. 27% in controls; χ^2^ = 10.37; *p* = 0.001; *n* = 444); no sex differences were found either in the control group (31.3% in women vs. 22.4% in men) nor in the AD group (69% in women vs. 69% in men).

When looking at neuropathologic characteristics, brain weight was lower in women (*p* < 0.001), even when corrected for age, while no other sex differences were found for Braak stage, plaque density, total brain NF or plaque load in the overall group. In the control group only, plaque density was higher in women than in men (*p* = 0.038), but was not significant after age correction (*p* = 0.066).

### 3.1. Depression as measured by the HAM-D

**Figure 1:**
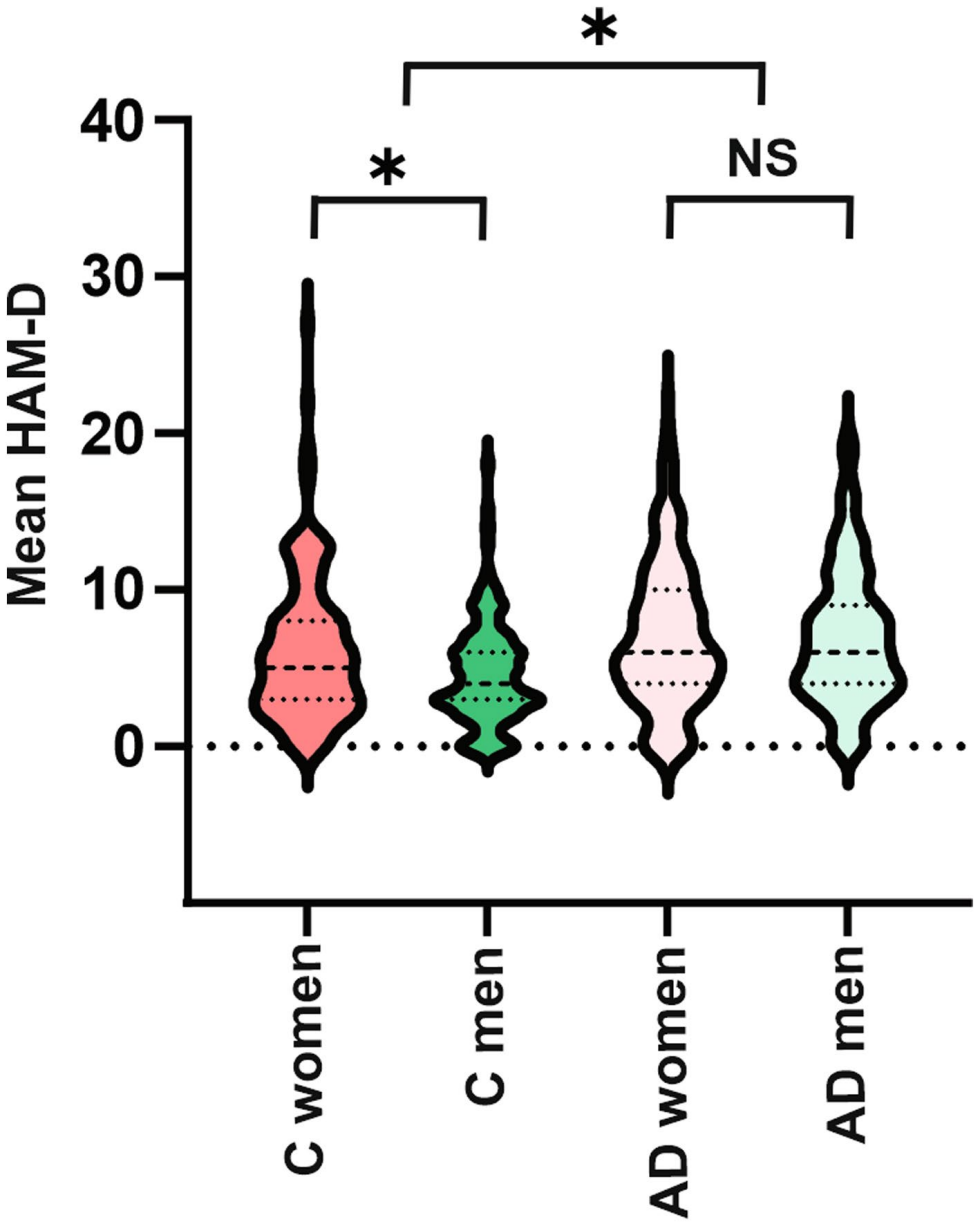
Violin plots of HAM-D depression scores between each group and sex. Sex difference is found only in controls, with a higher severity of depression in control women than men. No sex differences are found in AD. AD subjects have higher depression severity than controls. C = controls, AD = Alzheimer’s disease, HAM-D = Hamilton Rating Scale for Depression.

**Figure 2:**
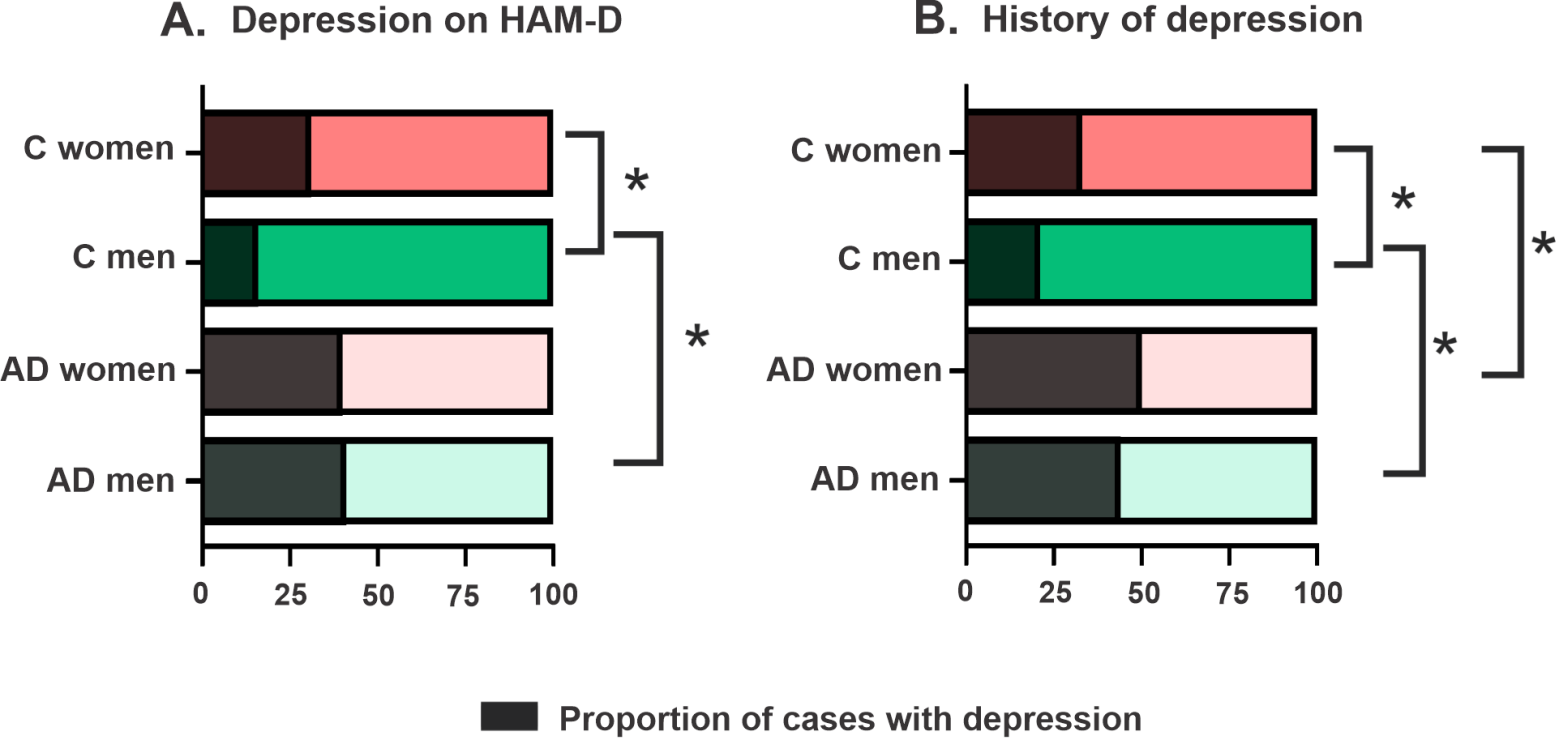
Proportions of subjects with depression in each group and sex. **(A)** When depression is defined by the HAM-D, proportion of cases with depression is higher in control women than control men and in control men than in AD men. **(B)** When depression is defined with history of depression and treatment with anti-depressant, depression is higher in control women than control men or AD women and in control men than in AD men.

**Figure 3:**
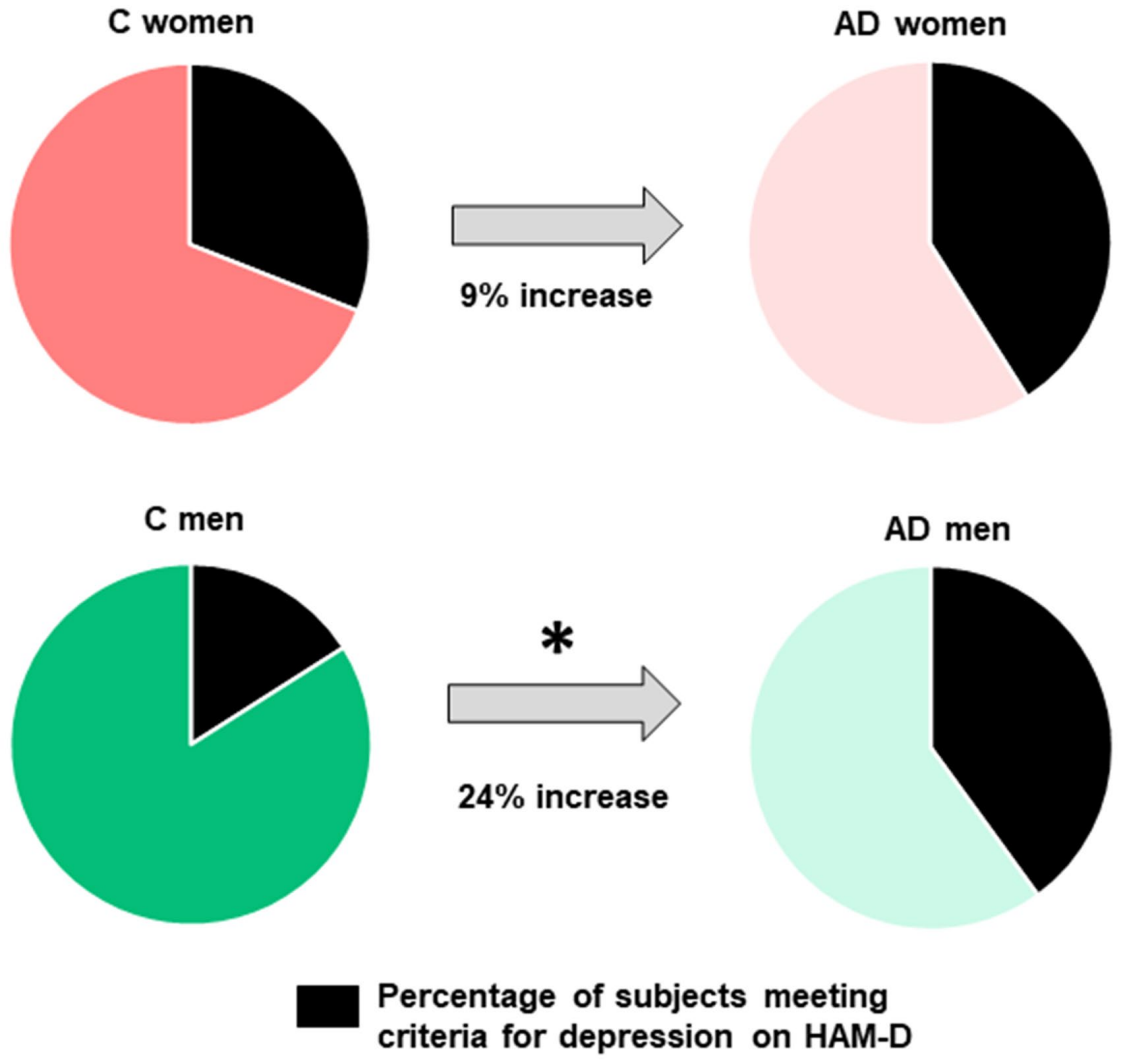
Increase between control and AD subjects in the proportion of subjects with depression. The increase in frequency of depression in controls versus AD is significantly greater in men (AD men - control men: 24%) than in women (AD women - control women: 9%).

In the whole group, 31% of subjects met criteria for depression on the HAM-D, of these 92.4% met criteria for mild depression, 7% met criteria for moderate depression and 0.5% met criteria for severe depression. Group differences were observed, as AD subjects had higher mean HAM-D scores (7.0 ± 4.5 vs. 5.5 ± 4.1; U = 33.04; *p* < 0.001) and were more likely to meet cutoff scores for depression than controls (40.7 vs. 24.1%; χ^2^ = 17.7; *p* < 0.001). The proportion of women meeting the cut-off score for depression (105/286 = 36.7%) was not significantly different than the proportion of men (93/304 = 30.6%; χ^2^ = 2.48; *p* = 0.115) and no significant sex differences were found in mean HAM-D scores (6.7 ± 4.7 vs. 6.0 ± 4.2; U = 46.05; *p* = 0.211) in all subjects for both groups. However, when looking separately in control and AD groups, sex differences in depression were observed in the control group, with higher mean HAM-D scores in women (6.2 ± 4.7 vs. 4.7 ± 3.3; U = 9360.0; *p* = 0.018) and a higher proportion of women with depression (31.5%), when compared to control men (16.3%; χ^2^ = 8.06; *p* = 0.0045). In the AD group, no sex differences, in either mean HAM-D score (U = 14.07; *p* = 0.9) or proportion of subjects with depression (χ^2^ = 0.017; *p* = 0.89), were observed between women (41.0%) and men (40.3%; Figures 1, 2A). A trend was observed but no significant difference was reported between control women and women with AD (χ^2^ = 2.75; *p* = 0.09) while a higher proportion of men with AD, when compared to control men, had depression (χ^2^ = 19.98; *p* < 0.001). When comparing the increase in frequency of depression in controls versus AD the difference was significantly greater in men (AD men – control men: 24.0%) than in women (AD women – control women: 9.5%; χ^2^ = 6.95; *p* = 0.008; Figure 3).

In the control group, 35% of cases (49.4% of women, 50.6% of men) were found to have MCI. Of these, 24.7% met criteria for depression on the HAM-D, no gender differences were found in the proportion of cases with MCI (20.4% in men vs. 28.9% in women; χ^2^ = 0.85; *p* = 0.36). Moreover, no differences in proportions of cases with depression were observed between control cases with MCI (24.7%) and cognitively normal controls (23.1%; χ^2^ = 0.082; *p* = 0.77) while depression was more frequent in AD cases than in control-MCI cases (χ^2^ = 14.90; *p* = 0.0001).

Mild univariate correlations were found between HAM-D score and age at death (Rho = −0.100; *p* = 0.015), MMSE (Rho = −0.190; *p* < 0.001), age of onset of dementia (Rho = −0.180; *p* < 0.001), brain weight (Rho = −0.143; *p* < 0.001), number of major neuropathological diagnoses (Rho = 0.190; *p* < 0.001), Braak NF stage (Rho = 0.099; *p* = 0.016), plaque density (Rho = 0.120; *p* = 0.003), total brain NF load (Rho = 0.104; *p* < 0.012) and total brain plaque load (Rho = 0.151; *p* < 0.001) but HAM-D score did not correlate with years of education or duration of dementia. For subjects with more than one depression scale available, the HAM-D was correlated to the GDS depression score (Rho = 0.430; p < 0.001; *n* = 409), the depression severity (Rho = 0.265; *p* < 0.001; *n* = 439) and apathy severity (Rho = 0.241; *p* < 0.001; *n* = 439) of the NPI-Q. In controls, HAM-D was only correlated with brain weight (Rho = −0.218; *p* < 0.012). In the AD group only, Ham-D correlated with age at death (Rho = −0.161; *p* < 0.001) and age of onset of dementia (Rho = −0.180; *p* = 0.001).

In the overall group, subjects with depression were younger (*p* = 0.017), and, when adjusted for age and sex, they were more likely to have a lower MMSE score (*p* < 0.001), a higher number of major neuropathological diagnoses (*p* = 0.001) and a lower brain weight (*p* < 0,001), as well as a higher Braak NF stage (*p* = 0.025), plaque density (*p* < 0.0001), total brain NF load (*p* = 0.011) and total brain plaque load (*p* < 0.0001). However, when investigating the controls, only brain weight (*p* = 0.014) was lower in cases with depression, but no significant differences survived age and sex correction. In the AD group, subjects with depression were younger but no other differences were observed. Further, when correcting for group (AD vs. control) none of the neuropathologic characteristic significantly predicted depression.

Further, logistic regression modeling found a lower MMSE score (*p* = 0.005) and female sex (*p* = 0.05), but not age or number of major neuropathologic diagnoses, to be significant predictors of depression [χ^2^ (4) = 26.84; *p* < 0.001; R^2^ = 0.062; for model]. In a model excluding all subjects with a second major neuropathologic diagnosis other than AD, i.e., including controls and subjects with AD diagnosis solely, both female sex (*p* = 0.03) and MMSE (*p* < 0.001) remained significant independent predictors of depression [χ^2^ (3) = 23.12; *p* < 0.001; R^2^ = 0.078; for model]. When adding Braak NF stage, plaque density, total NF tangle and plaque brain load the model was still significant [χ^2^ (7) = 27.86; *p* < 0.001; R2 = 0.095], with sex (*p* = 0.034), MMSE (*p* = 0.002) and higher total plaque load (*p* = 0.016) being significant independent predictors of depression.

When investigating controls only, female sex was found to be the only independent predictor of depression, with covariates for age, MMSE, Braak stage and plaque density [χ^2^ (5) = 11.63; *p* = 0.04; R^2^ = 0.068]. A model including the AD group only significantly predicted depression [χ^2^ (4) = 11.92; *p* = 0.018; R^2^ = 0.048] with younger age being the only independent predictor of depression (*p* = 0.009).

Refer to Table 3A for significance, odd ratios (OR) and 95% confidence interval (CI) of each predictor for different models.

**Table 3:** Multiple logistic regression modelling to determine predictors of depression.

Predictors	*p*-value	Odds ratios	95% CI
A: With depression (dependent variable) defined by a cut-off score on the HAM-D.			
All subjects			
Equation	**<0.001**		
Sex	**0.05**	0.70	0.49, 1.00
Age	0.07	0.98	0.95, 1.00
MMSE	**0.005**	0.97	0.95, 0.99
Nb nptx dx	0.3	1.13	0.92, 1.38
Excluding subjects with a second major nptx dx (including controls and subjects with AD solely)			
Equation	**<0.001**		
Sex	**0.03**	1.683	1.05, 2.54
Age	0.9	1.00	0.97, 1.03
MMSE	**< 0.001**	0.909	0.87, 0.94
Adding AD pathology			
Equation	**<0.001**		
Sex	**0.034**	1.63	1.05, 2.54
Age	0.63	1.00	0.97, 1.03
MMSE	**0.002**	0.95	0.92, 0.98
Braak NF stage	0.69	0.94	0.74, 1.18
Plaque density	0.12	0.73	0.88, 1.38
NF load	0.65	0.98	0.90,1.07
Plaque load	**0.016**	1.11	1.02, 1.21
In control subjects only			
Equation	**0.02**		
Sex	**0.003**	2.56	1.37, 4.79
Age	0.3	1.02	0.97, 1.06
MMSE	0.4	0.96	0.84, 1.09
Braak NF stage	0.2	0.77	0.54, 1.10
Plaque density	0.7	1.0	0.82, 1.35
In AD subjects only			
Equation	**0.02**		
Sex	0.72	1.09	0.69, 1.72
Age	**0.009**	0.96	0.93, 0.99
MMSE	0.19	0.98	0.96, 1.00
Nb nptx dx	0.60	0.92	0.67, 1.26
B: With depression (dependent variable) reported in a subject’s medical history combined with a treatment with antidepressants.			
All subjects			
Equation	**<0.001**		
Sex	**0.02**	1.51	1.07, 2.15
Age	**0.004**	0.94	0.94, 1.00
MMSE	0.181	0.96	0.96, 1.00
Nb nptx dx	**0.001**	1.39	1.14, 1.70
Excluding subjects with a second major nptx dx (including controls and subjects with AD solely)			
Equation	**0.002**		
Sex	**0.024**	1.64	1.07, 2.51
Age	**0.048**	0.97	0.94, 1.00
MMSE	**0.012**	0.97	0.94, 0.99
Adding AD pathology			
Equation	**0.026**		
Sex	**0.034**	1.60	1.04, 2.46
Age	**0.034**	0.97	0.94, 0.99
MMSE	0.217	0.98	0.95, 1.00
Braak stage	0.870	0.92	0.83, 1.30
Plaque density	0.260	1.24	0.89, 1.80
NF tangle load	0.519	1.03	0.95, 1.11
Plaque load	0.404	0.96	0.89, 1,05
In control subjects only			
Equation	0.189		
Sex	**0.03**	1.92	1.07, 3.45
Age	0.25	0.97	0.93, 1.01
MMSE	0.14	0.92	0.81, 1.04
Braak stage	0.43	0.90	0.62, 1,22
Plaque density	0.92	1.0	0.80, 1.28
In AD subjects only			
Equation	**0.034**		
Sex	0.16	1.38	0.88, 2.16
Age	**0.006**	0.93	0.93, 0.99
MMSE	0.94	0.99	0.97, 1.03
Nb nptx dx	0.35	1.16	0.67, 1.26

MMSE = last Mini Mental State Examination score; Nb nptx dx: number or major neuropathologic diagnoses. Depression was used as the dependent variable, subjects with depression were coded as 1 and subjects without depression as 0. Female sex was coded as 1 while male sex was coded as 0. Significant *p* values are in bold.

### 3.2. Depression reported in medical history combined with treatment with antidepressants

In the overall group, 38.4% of subjects had both a medical history of depression and received treatment with anti-depressants. A previous diagnosis of depression was significantly more frequent in AD subjects (47%) than in control subjects (27.1%; χ^2^ = 24.51; *p* < 0.001) and depression was more frequent in AD women (49.7%) than in control women (32.8%; χ^2^ = 8.33; *p* = 0.004) as well as in AD men (44.8%) when compared to control men (21.3%; χ^2^ = 17.35; *p* < 0.001). In the overall group, no significant statistical difference was reported in the frequency of depression reported by women (41.7%) and men (35.4%; χ^2^ = 2.48; *p* = 0.11), when excluding subjects with a second neuropathologic diagnosis, this sex difference was significant (χ^2^ = 4.37; *p* = 0.04). Similar to the results yielded using the HAM-D, a sex difference was observed when investigating the control group only, in which a higher proportion of women (32.8%) had depression when compared to men (21.3%; χ^2^ = 4.06; *p* = 0.044) but no statistical differences were found between women (49.7%) and men (44.8%) with AD (χ^2^ = 0.82; *p* = 0.37). The increase in frequency of depression in controls versus AD was not significantly different in men (AD men – control men: 23.5%) than in women (AD women – control women: 16.9%; χ^2^ = 1.50; *p* = 0.22; Figure 2B).

Of control cases that had MCI, 30.3% had an history of depression, no significant gender differences were found in the proportion of cases with MCI (25.0% in men vs. 35.6% in women; χ^2^ = 0.173; *p* = 0.28). Moreover, no differences in proportions of cases with depression were observed between control cases with MCI (30.3%) and cognitively normal controls (25.4%; χ^2^ = 0.701; *p* = 0.4) while depression was more frequent in AD cases than in control-MCI cases (χ^2^ = 7.99; *p* = 0.004).

Subjects with depression were younger (*p* < 0.001), and, when adjusted for age and sex, they were more likely to have a lower MMSE score (*p* < 0,001), a worse HAM-D score (p < 0,001) and GDS (*p* < 0,001) depression score, a greater number of major neuropathological diagnoses (*p* = 0.001) as well as a higher Braak NF stage (*p* = 0.005), plaque density (*p* = 0.002), total brain NF load (*p* = 0.04) and total brain plaque load (*p* = 0.014). However, when investigating the control group only, no significant differences survived age and sex correction. In the AD group, subjects with depression were younger but no other group differences were observed. Further, when correcting for group (AD vs. control) none of the neuropathologic characteristic significantly predicted depression.

Moreover, logistic regression modelling significantly predicted the presence of depression [χ^2^ (4) = 38.97; *p* < 0.001; R^2^ = 0.087], with female sex (*p* = 0.02), younger age (*p* = 0.004) and a higher number of major neuropathologic diagnoses (*p* = 0.001), but not MMSE, as significant independent predictors of depression. When excluding all subjects with a second major neuropathologic diagnosis, the model still predicted the presence of depression [χ^2^ (3) = 15.14; *p* = 0.002; R^2^ = 0.051], with sex (*p* = 0.024;), age (*p* = 0.048) and a lower MMSE (*p* = 0.012) being significant independent predictors. When adding Braak NF stage, plaque density, total NF tangle and plaque brain load, the model was still significant [χ^2^ (7) = 15.93; *p* < 0.026; R^2^ = 0.054] with the only independent predictors of depression remaining female sex (*p* = 0.034) and younger age (*p* = 0.034). In control only, the model was not significant [χ^2^ (5) = 7.45; *p* = 0.189; R^2^ = 0.042], while in the AD only group the model significantly predicted depression [χ^2^ (4) = 10.42; *p* = 0.034; R^2^ = 0.041] and younger age was the only significant independent predictor (*p* = 0.006; Table 3B).

## 4. Discussion

This clinicopathological study investigated sex differences in depression in pathologically well-defined subjects with AD dementia as well as non-demented controls. We confirm that AD dementia was associated with higher rates of depression than controls both when measured using a validated scale and by history of depression combined with anti-depressant treatment. Our results demonstrate that women had a higher likelihood of depression than men, but this sex difference was not observed when considering only those with AD dementia. Further, the increase in rates of depression, in AD subjects in comparison to controls, was greater in men than in women. These differences emphasize the importance of studying sex differences in AD and can have important implications for management of depression in AD, including more frequent depression screenings particularly in men who may be less likely to self-report depressive symptoms.

Depression has been shown to affect a large number of subjects with AD, we also report increased frequency of depression in AD affecting 40 to 47% of subjects (measured by HAM-D and history of depression respectively) when compared to 24 to 27% of subjects affected by depression in controls. A higher prevalence and severity of symptoms of depression have been repeatedly reported in women when compared to men (Parker and Brotchie, 2010; Eid et al., 2019). In our sample, the proportion of all women versus all men with depression did not reach statistical significance (36.7 to 41.7% in women and 30.6 to 35.4% in men) and we did not observe sex differences in severity of depression as measured by the HAM-D scores in the whole group. However, in logistic modelling, female sex was found to be a significant independent predictor of depression when controlling for age and cognitive status as measured by the MMSE. This effect remained when excluding subjects with a second major neuropathologic diagnosis, to account for the effect of multiple brain pathologies, and when controlling for AD neuropathology. When looking in both groups separately, we found that this sex difference observed was driven by the non-demented control group that showed a higher proportion of women with depression and more severe HAM-D scores in women than in men, while no sex differences were detected when considering only the AD group. It is possible that women have greater vulnerability to network disruptions or neurochemical imbalances that lead to mood symptoms in prodromal stages and reach a ceiling affect once they reach dementia stages. Future studies should correlate depression in women with biological markers of AD pathology such as serum amyloid beta, phosphor-tau, CSF and imaging markers.

While most studies report sex differences in depression, when investigating AD specifically, inconsistencies regarding sex differences in depression (Lovheim et al., 2009; Lee et al., 2017; Tao et al., 2018; Eikelboom et al., 2022) as well as the influence of sex in the association of depression as a risk factor for AD have been reported (Underwood et al., 2019; Zhu et al., 2021). These inconsistent results in AD may be related to the challenges of diagnosing depression in AD, as symptoms of depression and dementia overlap and there is no clear consensus to diagnose depression in AD (Burke et al., 2019). This highlights the need for more accurate tools to diagnose depression in AD. Moreover, these studies frequently lack pathological or biomarker confirmation of AD diagnosis which may also affect the results as distinct sex differences were reported in other frequent comorbid pathologies, such as Lewy body pathology, and sex differences in depression may present differently in other diseases affecting the brain (Perrin et al., 2017; Devanand et al., 2022; Chiu et al., 2023). Several hypotheses may be put forward as to why we do not find sex differences in AD in our study. Interestingly, when contrasting AD to non demented controls, we found that the AD-control increase in frequency of cases with depression (using the HAM-D score) was significantly greater in men (24%) than in women (9%). This result might suggest that men may be more likely to report or be diagnosed with depression when clinically affected with AD. One can speculate that these results could link to a gender bias in diagnosing mood disorders, in which men would be less likely to be diagnosed with a mood disorder in the absence of other medical conditions, such as dementia here (Norman, 2014; Salk et al., 2017). Several other neuropsychiatric symptoms such as apathy also frequently manifest in AD dementia and it may be difficult to separately assess depression from other symptoms (Eikelboom et al., 2022). While apathy measures were available only for a subset of subjects, no sex differences in apathy frequency or severity were found in either the AD or control group. Some of the neuropsychiatric manifestations, such as disinhibition in particular, to also play a role in this observed difference between men control and men with AD. It could be hypothesized that if men lose their inhibition when developing dementia this could lead to them, or their caregivers, reporting more symptoms of depression. Future studies should investigate the possible impact of disinhibition on the increase of depressive symptoms of depression in men. Alternatively, biological sex differences in depression have been reported and women have been shown to present with higher levels of inflammatory, neurotrophic, and serotonergic markers that were correlated to severity of depressive symptoms (Labaka et al., 2018). Future studies could further investigate potential sex differences in depletion of serotonergic, dopaminergic, or noradrenergic cortical afferents between men and women with AD.

Depression has been suggested to be an early manifestation of AD; depressive symptoms have been associated with an increased risk of developing dementia and depression is a predictor of progression from normal cognition to MCI and to dementia (Panza et al., 2010; Barnes et al., 2012; Hudon et al., 2020; Saiz-Vazquez et al., 2021). Consequently, depression was suggested to be a manifestation of AD biological process, sometimes preceding cognitive decline. The concept of Mild Behavioral Impairment (MBI) describes this trajectory of depression and/or other debilitating neuropsychiatric symptoms preceding the onset of AD dementia symptoms (Ismail et al., 2016). In our group, only a low number of cases had MCI and we found no differences in depression in control cases that had MCI when compared to cognitively normal controls. Overall, we found subjects with depression to be younger and more likely to have a lower MMSE score, as well as a greater level of AD neuropathology both in terms of NF tangles and neuritic plaque burden, when compared to subjects without depression. Previous studies also reported a more severe NF pathology in depression with co-morbid AD (Rupp et al., 2008) and early neurofibrillary pathology was associated to increased odd for depression and other neuropsychiatric symptoms (Ehrenberg et al., 2018). Accordingly, NF tangles affect noradrenergic and serotonergic brainstem neurons early in AD (Braak et al., 2006; Grudzien et al., 2007). However, the literature has yielded mixed results and in a larger study, using data from the National Alzheimer’s Coordinating Center, neuropathology was not associated with depression in subjects that had died with MCI and early AD dementia (McCutcheon et al., 2016). Our result might be linked to higher proportions of AD cases with depression as it is important to note that we did not find any differences in AD neuropathology when considering only the AD or only the control group and neither tau nor amyloid burden significantly predicted depression in controls. These results suggest that AD neuropathology markers do not explain the sex differences observed in the proportions of cases with depression found in controls and that depression in healthy aging is independent of NFT and Amyloid pathology, at least when measured using whole brain burden.

Several sex differences were reported in large pathologically well-defined cohort of AD suggesting a higher clinical and pathological severity in women including a higher neurofibrillary burden and a greater AD – Control brain weight loss (Filon et al., 2016; Liesinger et al., 2018; Barnes et al., 2019). We report an older age of onset of dementia and age of death in women as well as a lower brain weight while MMSE and other neuropathologic characteristic were similar between the sexes when controlled for age, but this is probably a result of having a smaller sample size in this study. A better understanding of sex differences in AD will lead to better recognition of risk factors of AD and better therapeutic management of patients.

We acknowledge some limitations. This study examined cross-sectional relationships between AD neuropathology and depression before death and causal inferences cannot be drawn. Depression had been reported to be recurrent and the use of medication might have improved the score on the HAM-D in well-controlled depression, thus, we used the worst HAM-D score when more than one test result was available, to capture any depression. Even though the HAM-D is a validated and widely used depression rating scale, it is designed as a semi-structured interview that requires a trained interviewer as well as a fair amount of judgement and interaction with the patient which makes this test likely to be an imperfect measure to use with demented patients (Cusin et al., 2009). Though, neuropsychologists and trained psychometrists routinely administers these tests in BBDP. Another limitation is that only one scale was used to assess depression, even though the GDS and NPIQ measures of depression were available for a subset of patients, it would significantly reduce the sample size and tests were not administered in the same time frame which would make it less likely to capture depression on two separate tests. Future studies using similar comparable measures would be more beneficial. Moreover, the use of medication is an important limitation of this study that should not be overlooked; a considerable number of subjects (38%) had received antidepressant medication that might have affected the test result. Information on depression treatment were limited, and subjects with dementia might have been prescribed less commonly used medications for depression such as mood stabilizers, while other types of treatment strategies such as cognitive behavioral therapy, might have not been captured in our dataset. To account for this, one strength of this study is that we used two complementary ways to define depression, both a validated clinician administered scale and history of depression in the subject’s medical history combined with treatment with anti-depressants, which have yielded similar results. Another possible confounder that wasn’t assess in this study is that the use of antidepressants may be associated with an increased risk of dementia (Wang et al., 2018; Kodesh et al., 2019).

In conclusion, in a large sample of subject with pathologically defined AD and controls, we found higher rates of depression in AD dementia. Female sex was a significant predictor of depression and women had a higher likelihood of depression than men in controls, while this difference was not noted when considering only AD subjects. This difference observed in controls was not explained by levels of AD neuropathology markers. The increase in rates of depression, in AD subjects in comparison to controls, was greater in men than in women which might suggest that men may be more likely to report or be diagnosed with depression when clinically affected with AD.

## Data availability statement

The raw data supporting the conclusions of this article will be made available by the authors, without undue reservation.

## Ethics statement

The studies involving human participants were reviewed and approved by Banner Sun Health Research Institute. The patients/participants provided their written informed consent to participate in this study.

## Author contributions

CT, PC, TB, and GS: conception and design. CT, PC, CB, IL, DG, TB, and GS: experimentations. CT and TB: statistical analysis, CT: writing—original draft preparation. CT, PC, CB, IL, DG, TB, and GS: writing—review and editing. All authors contributed to the article and approved the submitted version.

## Funding

The Arizona Study of Aging and Neurodegenerative Disorders and Brain and Body Donation Program has been supported by the National Institute of Neurological Disorders and Stroke (U24 NS072026 National Brain and Tissue Resource for Parkinson’s Disease and Related Disorders), the National Institute on Aging (P30 AG19610 and P30AG072980, Arizona Alzheimer’s Disease Center), the Arizona Department of Health Services (contract 211002, Arizona Alzheimer’s Research Center), the Arizona Biomedical Research Commission (contracts 4001, 0011, 05-901, and 1001 to the Arizona Parkinson’s Disease Consortium), and the Michael J. Fox Foundation for Parkinson’s Research.

## Conflict of interest

The authors declare that the research was conducted in the absence of any commercial or financial relationships that could be construed as a potential conflict of interest.

## Publisher’s note

All claims expressed in this article are solely those of the authors and do not necessarily represent those of their affiliated organizations, or those of the publisher, the editors and the reviewers. Any product that may be evaluated in this article, or claim that may be made by its manufacturer, is not guaranteed or endorsed by the publisher.
